# Hardening of shear band in metallic glass

**DOI:** 10.1038/s41598-017-07669-9

**Published:** 2017-08-01

**Authors:** J. G. Wang, Y. C. Hu, P. F. Guan, K. K. Song, L. Wang, G. Wang, Y. Pan, B. Sarac, J. Eckert

**Affiliations:** 1 School of Materials Science and Engineering, Southeast University, Nanjing, 210096 China; 2 Institute of Physics, Chinese Academy of Sciences, Beijing, 100190 China; 3 Materials and Energy Division, Beijing Computational Science Research Center, Beijing, 100193 China; 4 School of Mechanical, Electrical & Information Engineering, Shandong University (Weihai), Weihai, 264209 China; 5 Laboratory for Microstructures, Institute of Materials, Shanghai University, Shanghai, 200444 China; 6 Erich Schmid Institute of Materials Science, Austrian Academy of Sciences, Jahnstraße 12, A-8700 Leoben, Austria; 7 Department Materials Physics, Montanuniversität Leoben, Jahnstraße 12, A-8700 Leoben, Austria

## Abstract

Strain hardening, originating from defects such as the dislocation, avails conventional metals of high engineering reliability in applications. However, the hardenability of metallic glass is a long-standing concern due to the lack of similar defects. In this work, we carefully examine the stress-strain relationship in three bulk monolithic metallic glasses. The results show that hardening is surely available in metallic glasses if the effective load-bearing area is considered instantly. The hardening is proposed to result from the remelting and ensuing solidification of the shear-band material under a hydrostatic pressure imposed by the normal stress during the shear banding event. This applied-pressure quenching densifies the metallic glass by discharging the free volume. On the other hand, as validated by molecular dynamics simulations, the pressure promotes the icosahedral short-range order. The densification and icosahedral clusters both contribute to the increase of the shear strength and therefore the hardening in metallic glasses.

## Introduction

Strain hardening is a highly desired property for structural materials, especially for high-strength engineering metals and alloys^1^. It renders the strained material harder and increasingly difficult to deform further in the plastic regime, and therefore enables the material to accommodate the plastic strain globally. This prevents premature strain localization like necking in materials under tension and benefits the materials in terms of mechanical reliability. In general, strain hardening is ascribed to the multiplication, interaction or entanglement of dislocations in conventional crystalline metals^1^. On the contrary, no crystallographic defects like dislocation have been found to make the strain hardening available in monolithic metallic glasses (MGs) so far^2–6^. As a result, the plastic strain cannot be spread out in the whole sample; instead, it is often localized into thin shear bands in MGs at room temperature^7–9^. Worse yet, this localization is self-catalytic, so one primary shear band carries more and more strain and eventually develops into a crack causing the fracture^7^. One can easily infer that the lack of strain hardening must lead to a low damage tolerance in MGs. Partly for this reason, MGs are hardly employed in engineering applications despite their very high strength^10–12^.

For the MGs in which the plastic deformation proceeds via shear banding events^7^, their capability to be hardened substantially depends on the shear band itself^13^. Recently, Wang *et al*.^14^ have shown that the strain hardening is also possible in MGs. Stretching a Zr-based MG rod deeply notched, they found that the strain hardening indeed happened to the notched part. This hardening was attributed to densification as a net effect of free volume creation and annihilation. It must be noted that no shear band is developed in the plastically deformed material in ref. 10. If the shear bands has formed and developed during the plastic deformation of MGs, strain softening was frequently confirmed^15–18^. The softening was considered to result from shear-induced dilation and nano-voids formation and coalescence inside the shear bands^15, 17^. This notion has widely been accepted in the community^19, 20^. However, a work-hardenable Cu_47.5_Zr_47.5_Al_5_ MG was fabricated by Das *et al*.^21^. They achieved ~18% plasticity in the Cu_47.5_Zr_47.5_Al_5_ MG under compression. Scanning electron microscope (SEM) observation exhibited multiple branched and wavy shear bands on the surface of failed sample. It means the sample was not subject to a single dominant shear band. Conversely, a large number of shear bands carried a small fraction of strain each. This manner was associated with the atomic-scale inhomogeneity in the sample^21^. This kind of inhomogeneity has also been witnessed in other MGs, e.g. Zr_63.8_Ni_16.2_Cu_15_Al_5_^22^ and Zr_64.13_Cu_15.75_Ni_10.12_Al_10_^23^. More interestingly, in the very Zr_64.13_Cu_15.75_Ni_10.12_Al_10_ MG, Han *et al*.^24^ demonstrated that there was no strain hardening or strain softening at all, and the critical flow stress was invariant if the instant load-bearing area was taken into account. As such, a controversial issue arises: can the shear band in MG be hardened ever? This is not only a key problem of science but also directly related to the engineering application of MGs as mentioned above.

In this paper, we investigate the operating characteristics of shear bands in three typical Zr-based MGs through compression tests. The shear bands are found to harden indeed during the plastic deformation when the instant effective load-bearing area is considered. Even though the intrinsic shear strength is taken into account, the hardening is still apparently evident. However, the mechanism of hardening is significantly different from that in crystalline materials dominated by dislocations. Instead, we propose that remelting and solidification under a hydrostatic pressure imposed by the normal stress result in the hardening of the shear band material. Applying a hydrostatic pressure during the quenching process, molecular dynamics (MD) simulations reveal that the formed MG is densified as expected. More importantly, the atomic structure is remarkably tuned by the pressure, as identified by the Voronoi tessellation analysis. In particular, we find that the icosahedral clusters increase with the increasing applied pressure and they are responsible for the increase of shear strength in MGs.

## Results

### Microstructure investigation

**Figure 1:**
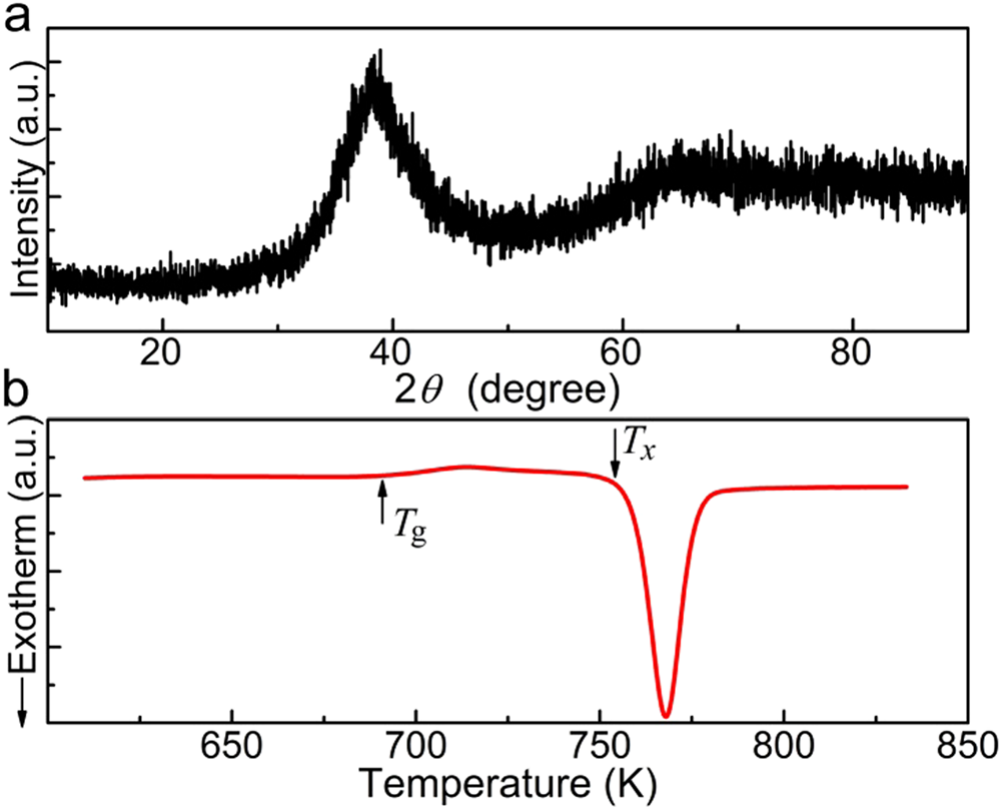
XRD pattern and DSC trace for Zr_50_Cu_44_Al_6_ MG. (**a**) The XRD pattern for the as-cast Zr_50_Cu_44_Al_6_ alloy shows broad and diffuse maxima, indicating its glassy nature. (**b**) The DSC (heating rate of 20 K/min) trace for the Zr_50_Cu_44_Al_6_ MG clearly presents the glass-transition marked by *T*_g_ and the crystallization onset marked by *T*_x_.

**Figure 2:**
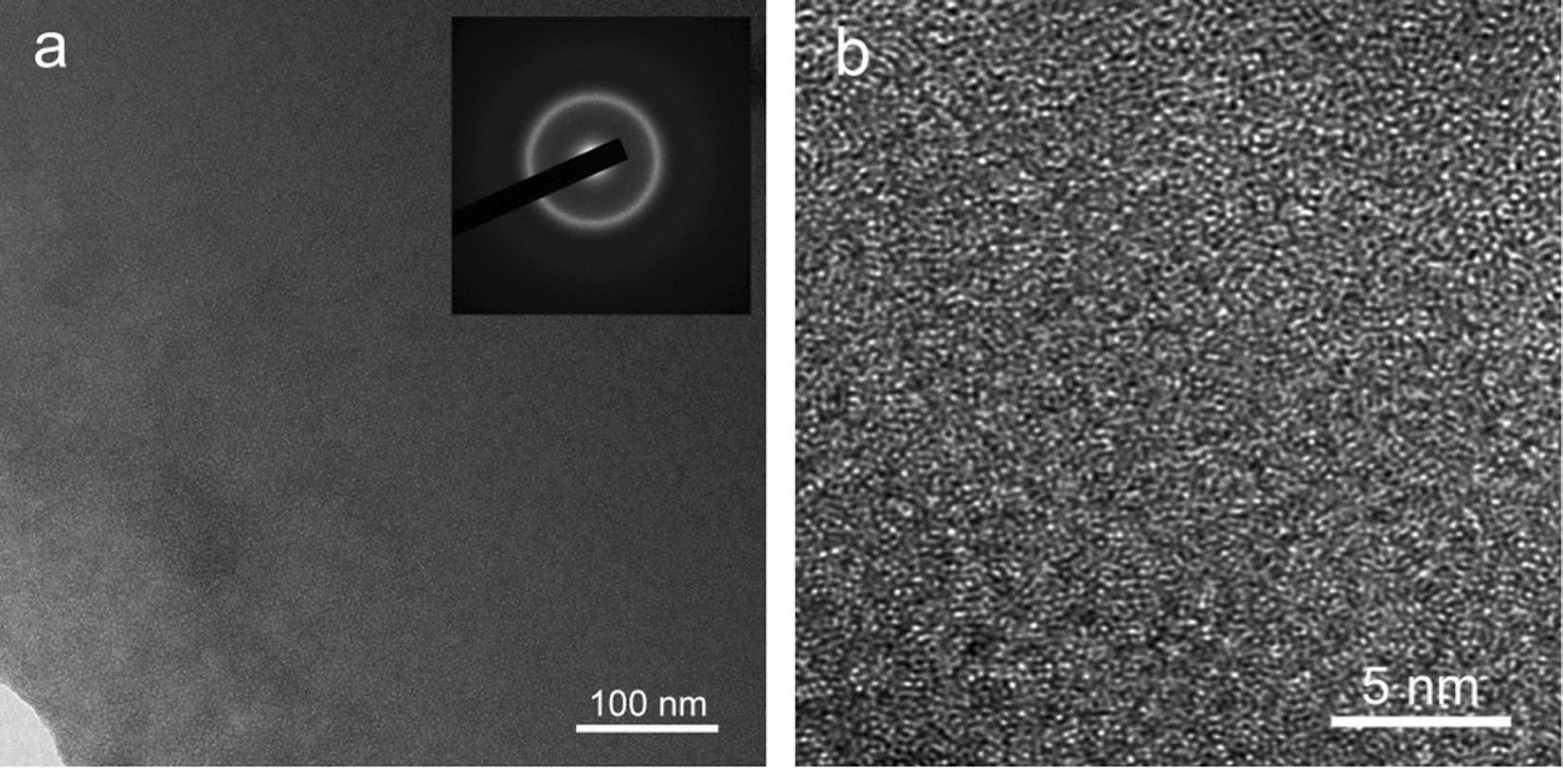
The amorphous microstructure of Zr_50_Cu_44_Al_6_ alloy. (**a**) TEM with a SAED pattern (inset) and (**b**) HRTEM.

To assure the MGs a fully glassy state, microstructure characterization and thermal analysis have been conducted. Figure 1 presents the XRD pattern and DSC trace for Zr_50_Cu_44_Al_6_. No any Bragg peaks can be seen in Fig. 1a, while the glass transition (marked by *T*_g_) and crystallization (marked by *T*_x_) behaviors are very clear in Fig. 1b. This implies the glass nature of Zr_50_Cu_44_Al_6_. Owing to their excellent glass forming ability, neither Zr_65_Cu_15_Ni_10_Al_10_ nor Vit 1 is subject to XRD and DSC^25, 26^. However, nanocrystals are sometimes observed to precipitate in the glassy matrix by TEM even though they are invisible to XRD technique^21, 27, 28^. Undoubtedly, these nanocrystals can tailor the properties of the sample. They may hamper the shear band generation and propagation in MGs, since the operation of dislocation and twinning inside the nanocrystals usually cause the strain hardening^27^. To rule out the presence of nanocrystals in the three MGs of this work, TEM are used. Figure 2a shows the TEM image for Zr_50_Cu_44_Al_6_ MG. The very homogeneous contrast indicates a glassy nature of the sample, which is consistent with selected area electron diffraction (SAED) pattern (the inset) exhibiting a diffuse halo ring. Moreover, lattice fringes corresponding to the crystalline phase cannot be seen in the high resolution TEM (HRTEM) image (Fig. 2b). It corroborates that the sample is a fully monolithic glass. Similar TEM results are obtained in Zr_65_Cu_15_Ni_10_Al_10_ (see Fig. S1) and Vit 1.

### Mechanical performance and the intrinsic shear strength

**Figure 3:**
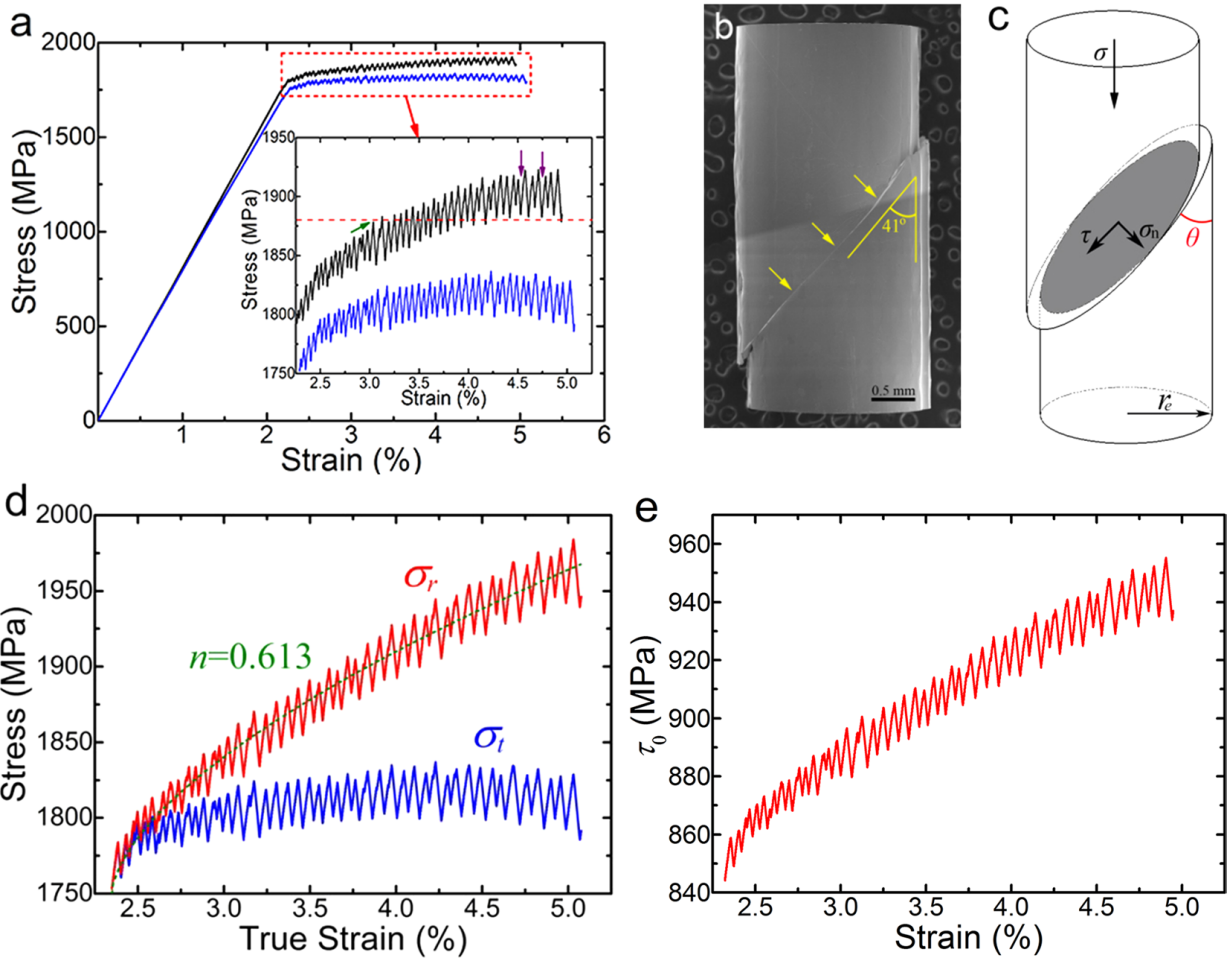
The investigation on the deformation behavior in Zr_50_Cu_44_Al_6_ MG under compression. (**a**) The engineering (black) and true (blue) stress-strain curves and the inset magnifying the plastic regime. (**b**) The SEM image of the deformed sample in which the primary shear band is marked by the arrows. (**c**) A schematic illustration of the effective load-bearing area in the rod sample. (**d**) The real stress (red) calculated using the area *A* in Eq. (2) showing the hardenability in Zr_50_Cu_44_Al_6_ MG with a strain hardening coefficient *n* = 0.613. (**e**) The intrinsic shear strength calculated by Eq. (4) shows a hardening behavior also.

Figure 3 presents the measurement and observation of the deformation behavior in the Zr_50_Cu_44_Al_6_ MG. In Fig. 3a, the true stress-strain curve (blue) is calculated according to the engineering one (black), in which the calculation is well known in textbooks as^1^:

1a $${\varepsilon }_{t}=\,\mathrm{ln}(1+{\varepsilon }_{e})$$

1b $${\sigma }_{t}={\sigma }_{e}(1+{\varepsilon }_{e})$$

where *ε*_*e*_, *σ*_*e*_, *ε*_*t*_ and *σ*_*t*_ are the engineering strain and stress and the true strain and stress, respectively. At the first glance, *σ*_*e*_ increases with the increasing *ε*_*e*_ in the plastic regime, which is clearer in the inset. In particular, the valley (marked by the violet arrow) of the serration in the latter part are higher than the peak (marked by the green arrow) for that in the first. It looks like the strain hardening. On the other hand, in the true stress-strain curve, stress almost keeps a constant with strain, which is in line with the conclusion by Han *et al*.^24^. However, the prerequisite for Eq. (1) that is used to calculate the true stress-strain is the constant volume of the sample^1^, which implies that the effective load-bearing area increases with the strain due to the decreasing length of the sample in the compression. Figure 3b shows the SEM image of the deformed sample. As marked by the arrows, only one primary shear band develops and accommodates all the plastic strain^29, 30^. More importantly, one can easily find that the load-bearing area is factually decreasing with the increasing strain, resulting in the invalidity of Eq. (1). As illustrated in Fig. 3c, the horizontal projected area of the instant load-bearing area (shaded region) with a shear angle *θ* is

2 $$A=2{r}_{e}^{2}(\phi -\,\sin \,\phi \,\cos \,\phi )$$

where $\phi =\arccos (\frac{{l}_{e}{\varepsilon }_{p}tg\theta }{2{r}_{e}})$, *r*_*e*_ and *l*_*e*_ are the radius and length of the rod sample at the elastic limit, and *ε*_*p*_ is the plastic strain. As a matter of fact, Han *et al*.^24^ adopted Eq. (2), but a fixed *θ* = 45° was taken. According to the statistics by Zhang *et al*.^31^, *θ* is a variable and usually less than 45° in compression, which holds true in this work (*θ* = 41°). Using *A* in Eq. (2), the real stress *σ*_*r*_ (red) is presented in Fig. 3d, significantly different from *σ*_*t*_ (blue). Obviously, *σ*_*r*_ increases with the plastic strain, which firmly demonstrates the characteristics of strain hardening. Along the Ludwik-Hollomon relationship^32^

3 $$\sigma ={\sigma }_{y}+k{\varepsilon }_{p}^{n}$$

where *σ*_*y*_, *k* and *n* are the yield stress, pre-exponential factor and strain-hardening coefficient, respectively, *n* = 0.613 is obtained for Zr_50_Cu_44_Al_6_ MG by fitting the stress-strain curve in plastic regime, similar to those for steel (0.36) and brass (0.42)^32^.

Unquestionably, shear band is operating in mode II. Therefore, the shear strength has attracted many attentions. Zhang *et al*.^31^ found that the measured shear stress *τ* depends on the normal stress *σ*_*y*_ in the shear plane as illustrated in Fig. 3c. Afterwards, Qu *et al*.^33^ formulated the *σ*_*y*_ dependence of the critical shear stress (or intrinsic shear strength) *τ*_0_ as

4 $${\tau }_{0}=\sqrt{{\tau }^{2}+{\alpha }^{2}\beta {\sigma }_{n}^{2}}$$

in which *α* = 2 (1 − 2*ν*)/(1 + *ν*) proposed by Liu *et al*.^34^, *ν* is Poisson’s ratio and *β* = −0.5 for compression. Figure 3e displays the plot of *τ*_0_ with *ε*_*p*_. Clearly, *τ*_0_ increases with increasing *ε*_*p*_, indicating the hardening of shear-band material. This also holds true in Zr_65_Cu_15_Ni_10_Al_10_ and Vit 1 MGs (see Figs S3 and S4). It should be noted that *σ*_*n*_ also increases with *ε*_*p*_, as shown in Fig. S2.

**Figure 4:**
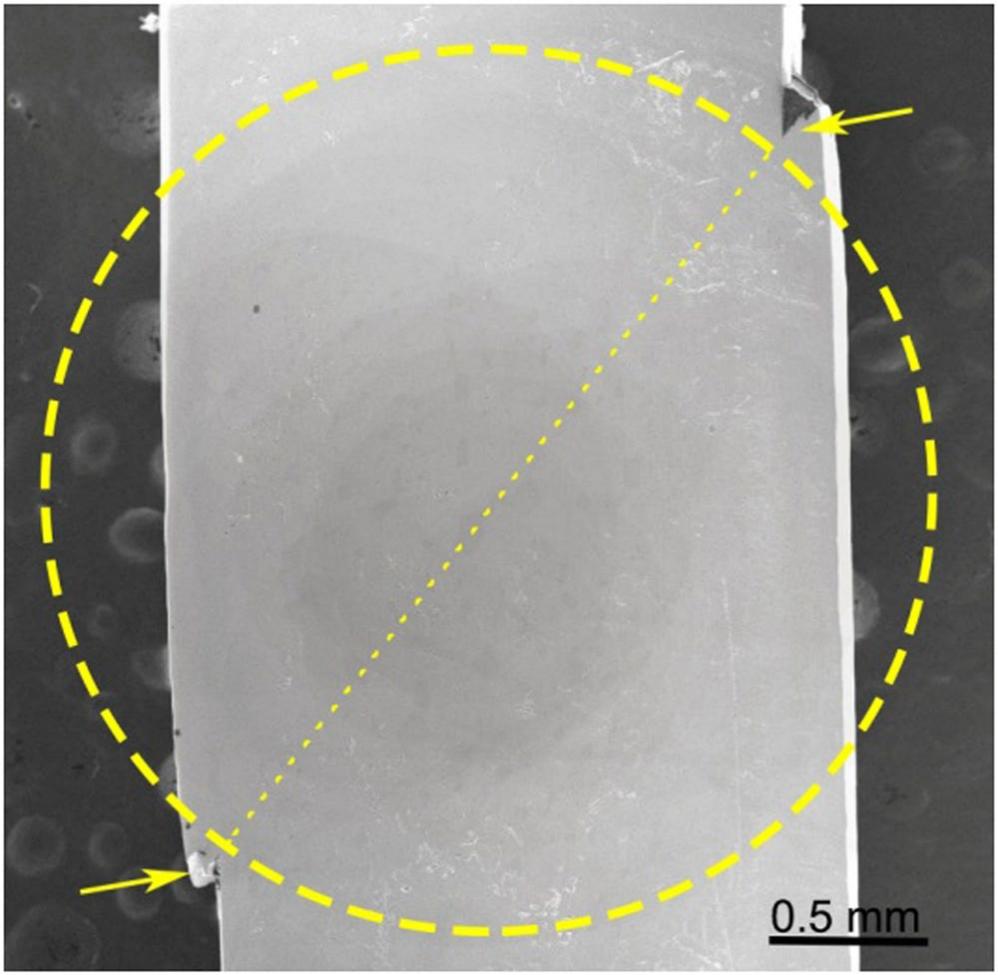
The SEM image of longitudinal section of the deformed sample shown in Fig. 2. The dotted line is expected to be the path of the shear band, and the region enclosed by the dashed circle is taken to prepare the TEM sample in Fig. S6.

Occasionally, inside the shear band precipitate some nanocrystallites in the deformed MG sample, though they are absent in the as-prepared sample^28, 35, 36^. The longitudinal section of the deformed sample in Fig. 3b is exhibited in Fig. 4. A region enclosed by the circle is taken as the TEM sample, in which the approximate path of shear band is marked by the dotted line. Then, the TEM observation is performed carefully, and the TEM imaging features (see details in Fig. S5) are extremely similar to those in Fig. 2. No any nanocrystallites can be found, which essentially excludes the involvement of dislocation-induced hardening in Fig. 3. In other words, there should be a different hardening mechanism in MGs.

### The role of hydrostatic pressure

Our previous studies showed that the temperature rise is significant inside the shear band during its sliding^37, 38^. Then they convincingly responded to the argument of no significant temperature rise^38–41^. Besides, the melted shear-band material is subsequently quenched at a ultrahigh cooling rate of ~10^8^ K/s at the end of a shear-banding event^37^. On the other hand, the hydrostatic pressure *P* imposed by the normal stress *σ*_*n*_ was found to play an important role, as suggested by Zhang *et al*.^31^. Generally, it has $P\approx \frac{2{\sigma }_{n}}{3}$ according to the spherical stress tensor^1^, and *P* increases due to the increasing *σ*_*n*_ during the plastic deformation (see Fig. S2-1). It implies that the effect of hydrostatic pressure will become greater and greater with the plastic strain. Herein, we do propose that the atomic structure of the shear-band material is remarkably mediated during the remelting and the following solidification under a hydrostatic pressure and therefore becomes more resistant to the shear. To validate this proposal, MD simulations were conducted for Zr_50_Cu_44_Al_6_ MG, but not for Zr_65_Cu_15_Ni_10_Al_10_ MG or Vit 1 owing to their inaccessible interaction potential so far.

**Figure 5:**
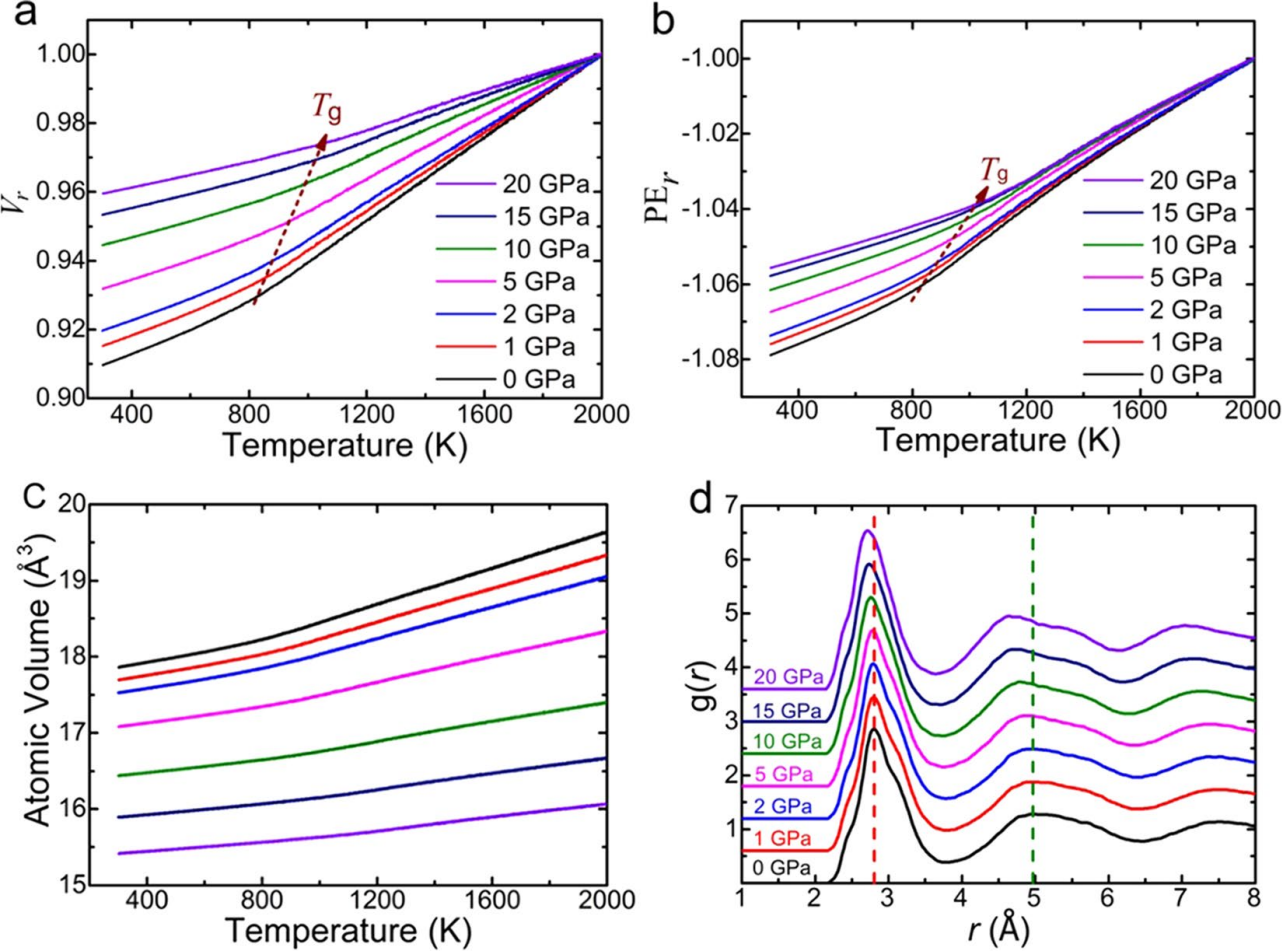
Description of glass transition of Zr_50_Cu_44_Al_6_ alloy in MD simulations. (**a**) The reduced volume *V*_*r*_ and (**b**) the reduced potential engergy *PE*_*r*_ against the temperature show an elevated glass transition temperature with the increasing hydrostatic pressure. (**c**) The atomic volume contracts at a certain temperature due to the applied hydrostatic pressure. In other words, the Zr_50_Cu_44_Al_6_ MG is densified, which is proved by the shift of the first and second peak position towards a smaller radial distance on the *g*(*r*) curve shown in (**d**).

Under a hydrostatic pressure, the glass transition happens at an elevated temperature in MGs^42^. Figure 5a and b show the reduced volume *V*_*r*_ = *V*/*V*_0_ and the reduced potential energy *PE*_*r*_ = *PE*/|*PE*_0_| (*V*_0_ and |*PE*_0_| are respectively the volume and the absolute value of potential energy at 2000 K) against the temperature under various pressures. Clearly, *T*_g_ increases with the applied the pressure, as marked by the dashed line. It is because the pressure can enhance the viscosity through reducing the free volume in the sample and therefore rendering the atomic diffusion sluggish^37^. As shown in Fig. 5c, the average atomic volume contracts greatly with the increasing pressure at a certain temperature. For instance, it is 17.9 Å^3^ for 0 GPa at 300 K, for 20 GPa with 14% decrement to15.4 Å^3^. Meanwhile, Fig. 5d shows that positions of the first and second peaks in pair correlation function *g*(*r*) shift to the smaller radial distance, indicating that the atoms come closer to each other. Obviously, the Zr_50_Cu_44_Al_6_ MG must be densified by the discharge of the free volume.

**Figure 6:**
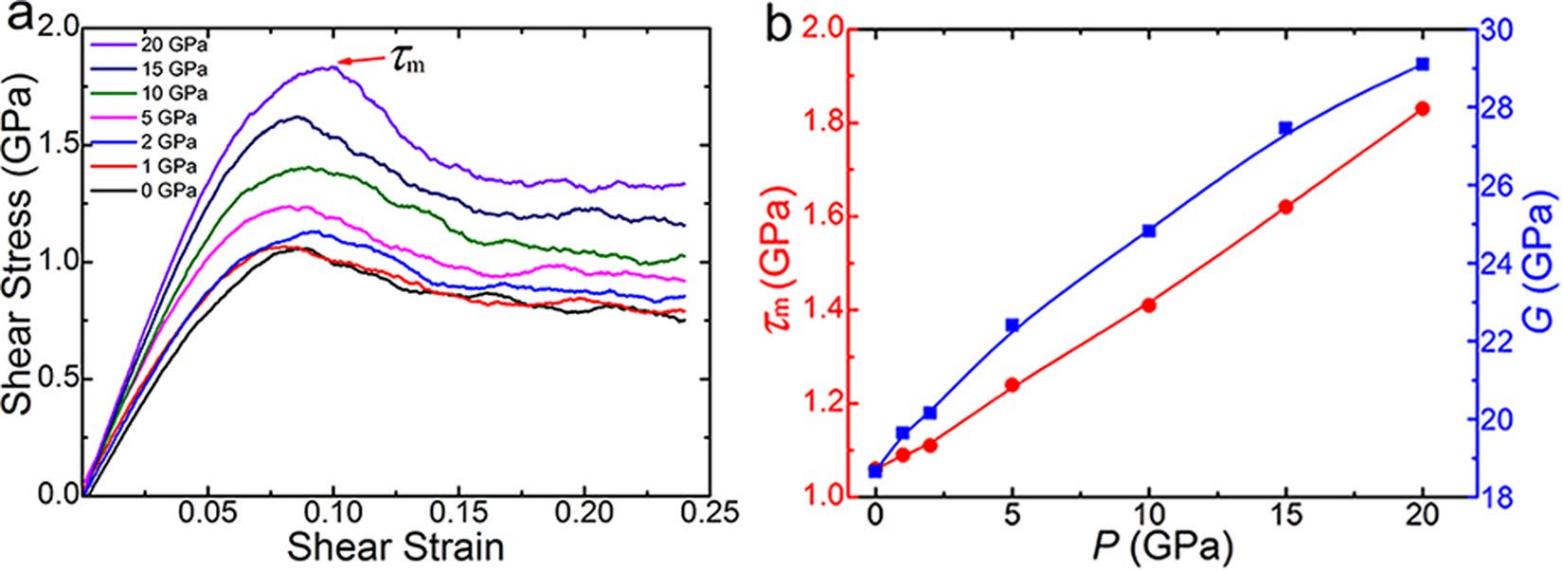
The MD simulations of the shear deformation in Zr_50_Cu_44_Al_6_ MG samples prepared under various hydrostatic pressures. (**a**) The shear stress-strain curves on which the maximum value *τ*_m_ is defined as the shear strength. (**b**) The shear strength and the shear modulus are enhanced by the increasing pressure.

The reduction of the free volume can always elevate the shear stress in MGs because of $\tau \propto \exp (\frac{C}{{v}_{f}})$ in which *C* is a constant for a given composition and *v*_*f*_ is the average free volume per atom^19^. Figure 6a shows the shear stress-strain curves for samples prepared under various pressures at 300 K. One can see that the shear strength increases with the increasing applied pressure. The shear strength here is defined as the peak value of the shear stress in the curve, i.e. *τ*_m_ marked in Fig. 6a. This trend is clearly presented in Fig. 6b. Moreover, the shear modulus *G* shares the same trend. Accordingly, it comes to a conclusion that the hardening presented in Fig. 3e should be caused by the remelting and solidification of shear-band material under a hydrostatic pressure. This effect is experimentally confirmed in a Zr_48_Cu_36_Al_8_Ag_8_ MG prepared though remelting and pressure-casting^43^.

### Atomic configuration

**Figure 7:**
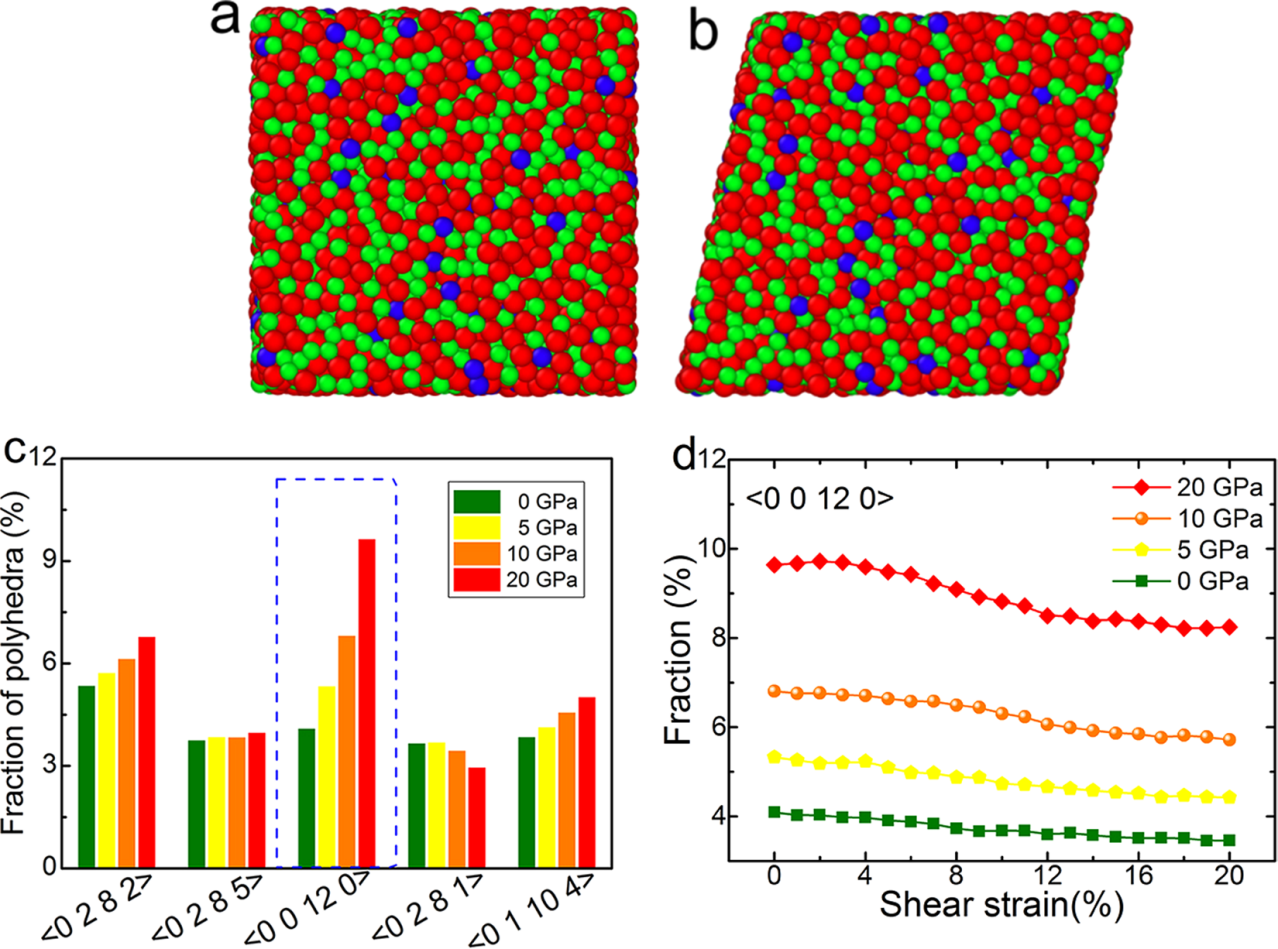
The examination on atomic structure of Zr_50_Cu_44_Al_6_ MG via MD simulations. The illustrations of (**a**) as-prepared and (**b**) 20% shear-strained atomic configurations in the simulation box with a size of about 52 × 52 × 52 Å^3^. (**c**) The fraction of Voronoi polyhedra of the five most common atom motifs in the as-prepared samples. (**d**) The fraction of icosahedron with a Voronoi index <0 0 12 0> varies with the increasing shear strain.

Based on the Voronoi tessellation analysis, the atomic structure has been examined in the as-prepared and deformed Zr_50_Cu_44_Al_6_ MG. Figure 7a and b schematically present the virginal and 20% shear-strained atomic configurations of Zr_50_Cu_44_Al_6_ MG, respectively. As shown in Fig. 7c, the first five commonest motifs in the virginal samples prepared under the applied pressure (only the results for 0, 5, 10 and 20 GPa are presented here) are <0 2 8 2>, <0 2 8 5>, <0 0 12 0>, <0 2 8 1> and <0 1 10 4>. The fractions of these motifs vary with the applied pressures. Along with the increasing pressure, <0 2 8 1> is subject to a slight decrease in fraction, while the four others grow with different increments. In particular, the fraction of <0 0 12 0> increases from 4.1% for 0 GPa to 9.6% for 20 GPa. In other words, the number of <0 0 12 0> is doubled in virtue of hydrostatic pressure. In fact, <0 0 12 0> represents the icosahedral-short-range-order which is a key feature of microstructure in MGs^6, 42, 44^. In simple liquids and MGs, icosahedron is proposed to be preferred even over the FCC and HCP packing due to its lower potential energy^44^. As a result, it needs to exert a larger stress to deform the icosahedra than to deform other clusters^45^. If the number of icosahedra increases in the sample, the yield stress of the material would be enhanced. This is reflected by Figs 6 and 7c. In addition, the evolution of these motifs is examined during the deformation of the sample. As shown in Fig. 7d, the fraction of icosahedra almost keeps constant within the first elastic strain of 4%. This is because the atomic bonding is not broken or rebuilt but just adjusted slightly in the elastic regime^46^. In the following strain range of 4% to 12%, the sample yields and deforms plastically, as presented in Fig. 6a. Correspondingly, Fig. 7d reveals that the fraction of icosahedra is reduced noticeably, indicating that icosahedra are disassembled or broken during the yielding and plastic deformation. After the strain of 12%, the fraction of icosahedron changes very slightly, but that for high pressure (e.g. 20 GPa) still remains higher than that for low pressure (e.g. 5 GPa). Interestingly, other motifs basically do not vary very much and almost keeps the original fraction (see details in Fig. S6). This proves that the icosahedra play a prominent role in the deformation of MG indeed.

## Discussion

However, if the hardening is available in the shear-band material as aforementioned, why do then not the secondary shear bands develop at other sites in the sample? In reality, the increasing stress is partly due to the decreasing load-bearing area as illustrated by the shaded region on the shear plane in Fig. 3c, so the shear stress at other sites is not increased so much as that on the shear plane. On the other hand, the first shear band must initiate somewhere with a relatively lower critical shear stress, say *τ*_1_, compared with that, say *τ*_2_, for elsewhere. A increment of critical shear stress, Δ*τ*, is caused by the hardening. If *A*(*τ*_1_ + Δ*τ*) < *A*_*e*_ *τ*_2_ in which ${A}_{e}=\pi {r}_{e}^{2}$, the secondary shear bands cannot be activated. This is the situation in present work. Once *A*(*τ*_1_ + Δ*τ*) > *A*_*e*_ *τ*_2_, the secondary and multiple shear bands are probably triggered, which has already been observed by a number of early studies^22, 23, 47^.

**Figure 8:**
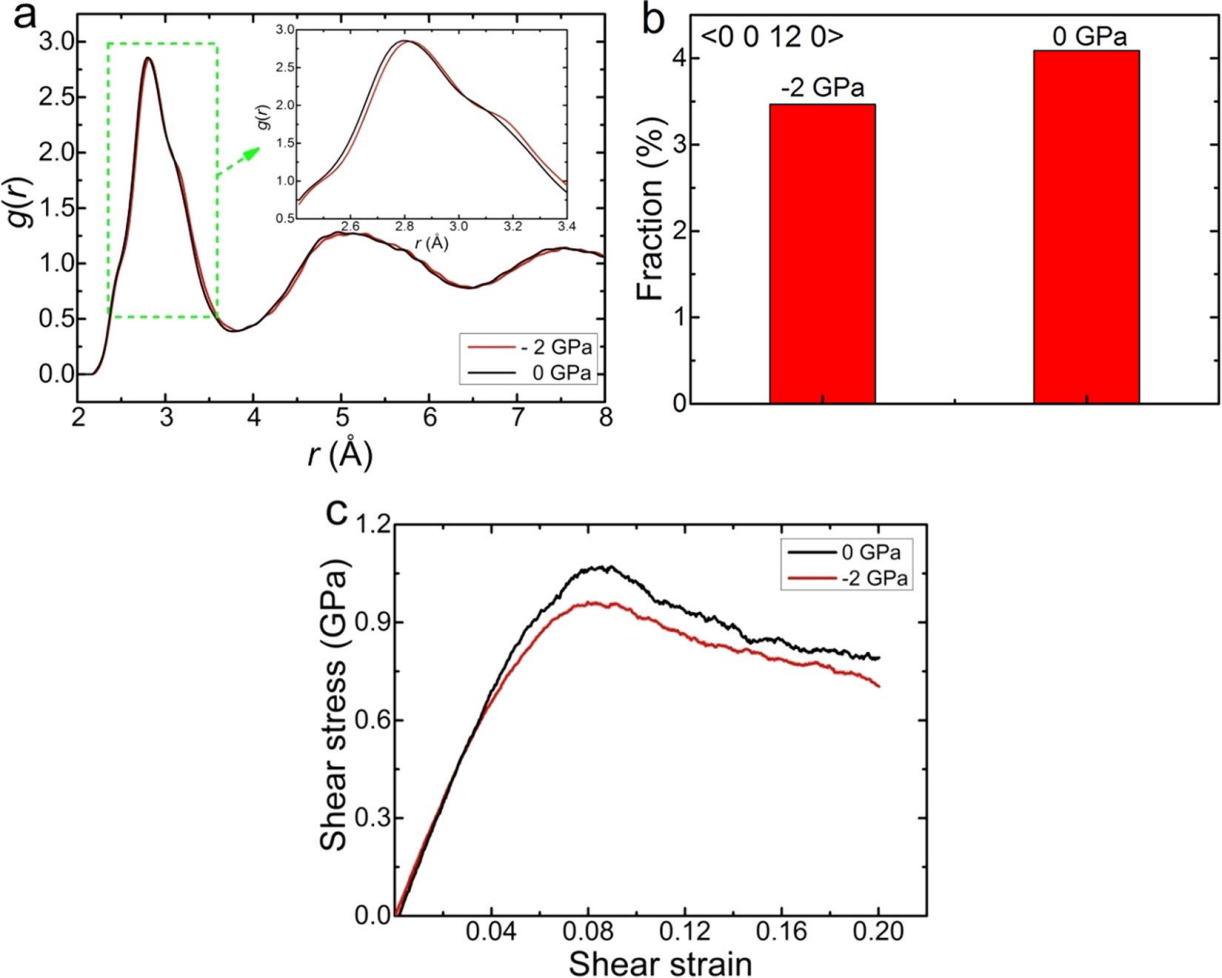
The MD simulation of Zr_50_Cu_44_Al_6_ MG under a negative hydrostatic pressure *P* = −2 GPa. (**a**) The *g*(*r*) curve on which the first peak position shifts to a larger radial distance for *P* = −2GPa (red) compared with that for *P* = 0 GPa (black). (**b**) The fraction of icosahedra indexed as <0 0 12 0> becomes less for *P* = −2 GPa than for *P* = 0 GPa. (**c**) The shear stength is reduced by the negative hydrostatic pressure at 300 K.

For the MGs under uniaxial tension, the multiple shear bands can hardly develop and only one primary shear band dominates always^48, 49^. As a matter of fact, the hydrostatic pressure *P* is negative inside the shear band under tension. Figure 8 shows the structural and deforming features in Zr_50_Cu_44_Al_6_ MG for *P* = −2 GPa compared with those for *P* = 0 GPa. As shown in Fig. 8a, the first peak in the *g*(*r*) curve shifts towards a larger radial distance *r*, indicating a lower packing density of atoms. Besides, the fraction of icosahedron characterized by the Voronoi index <0 0 12 0> is less for *P* = −2 GPa than for *P* = 0 GPa (Fig. 8b), consistent with the previous result of binary Cu-Zr MG^42^. Following the discussion above, the shear strength *τ*_m_ for *P* = −2 GPa should therefore be lower, which is actually verified in Fig. 8c essentially in agreement with the difference between the compressive and tensile strength of MGs^50, 51^. As such, the shear band will carry more and more shear strain and eventually develop itself to a crack. This explains the absence of tensile ductility in MGs. Nevertheless, it cannot help to understand the densification and strain hardening in the notched MG sample under tension in ref. 10 where there was no shear band found at all.

It is sometimes found that the stress drops with the strain even with consideration of Eq. (2), especially in the heavily deformed sample. The SEM observation shows that the micro-voids and/or cracks already appear in the shear band in the MG samples subject to the large plastic deformation^15, 17, 52^. They reduce the effective load-bearing area further and make Eq. (2) invalid. In this work, a small plastic strain less than 3% was therefore applied and no micro-voids or cracks is developed in the shear band as shown in Fig. 4, and therefore the hardening behavior is clearly presented in Figs 3, S3 and S4. Besides, it is sometimes found that the dilatation or cavitation happens to some very local sites in the shear band due to the internal tensile stress^53–55^. For a MG sample under compression, the shear band is globally subject to compressive stress instead of tensile one. As a result, the shear band must be densified and hardened as a whole, though some local regions may undergo the reverse.

In summary, we have investigated the deformation behavior in three monolithic metallic glasses. The hardening behavior is witnessed in the plastic regime when the effective load-bearing area is taken into account instantly. The hardening mechanism is proposed to originate from the remelting and solidification of the shear-band material under a hydrostatic pressure imposed by normal stress on the shear plane. As expected, the shear-band material is densified during the quenching, confirmed by the molecular dynamic simulations. Meanwhile, MD simulations show that the icosahedral clusters are favored by virtue of the pressure. The densification and icosahedra work together to enhance the strength of the metallic glass and therefore achieve the hardening. Our findings and analyses address the issue of the hardenability in metallic glasses to some degree. They help to understand the unique mechanical property more deeply and evaluate the engineering reliability of metallic glasses in applications.

## Methods

### Experimental procedure

Three typical MGs with nominal composition of Zr_50_Cu_44_Al_6_ (at.%), Zr_65_Cu_15_Ni_10_Al_10_ and Zr_41.2_Ti_13.8_Cu_12.5_Ni_10_Be_22.5_ (i.e. Vit 1) were prepared by arc-melting and then suction-casting pure metals into a water-cooled copper mold under a Ti-gettered argon atmosphere. The prepared rod-shaped MG samples have a diameter of 2 mm. Their glassy nature was ascertained by XRD method using BRUKER D8 ADVANCE and TEM of TECNAI-F20. Thermal analysis was performed using Perkin Elmer DSC-7 at a heating rate of 20 K/min. As-cast rod samples with an aspect ratio of around 2:1 were compressed using Instron 8562 machine at a strain rate of 10^−4^ s^−1^ at room temperature. In particular, great care was taken to ensure the two ends of sample for compression test flat and parallel to each other and perpendicular to the longitudinal loading axis. The samples were unloaded after ~5% total strain without fracture. The deformed samples were investigated by SEM of Philips XL30 instrument and TEM.

### MD simulations

In our work, MD simulations were performed to Zr_50_Cu_44_Al_6_ MG due to its accessible embedded-atom method (EAM) potential and details can be found in ref. 56. All the simulations were operated using the LAMMPS package^57^. The cubic box contains 8,000 atoms with periodic boundary conditions in three dimensions. In the preparation, the sample was first equilibrated at 2,000 K for 2 ns and then was quenched to 300 K at a cooling rate of 10^11^ K/s in the isobaric-isothermal (NPT) ensemble, during which the box size was adjusted to give the applied pressure. In the shear deformation process, the prepared sample was sheared at a strain rate of 10^8^ s^−1^ in the canonical NVT ensemble at 300 K. The atomic structure was characterized by Voronoi tessellation.

## Electronic supplementary material

Supplemental Materials
