# Supplementary material for: Hardening of shear band in metallic glass
Source: Sci Rep. 2017 Aug 1;7:7076. doi: 10.1038/s41598-017-07669-9 (PMC5539228; doi:10.1038/s41598-017-07669-9)
Supplement: Supplementary file 1 — Supplemental Materials [file 41598_2017_7669_MOESM1_ESM.pdf]

Supplemental Materials for

"Hardening of shear band in metallic glass"

J. G. Wang[1], Y. C. Hu[2], P. F. Guan[3], K. K. Song[4], L. Wang[4], G. Wang[5], Y. Pan[1], B. Sarac[6] & J. Eckert[6,7]

[1]School of Materials Science and Engineering, Southeast University, Nanjing 210096, China
[2]Institute of Physics, Chinese Academy of Sciences, Beijing 100190, China
[3]Materials and Energy Division, Beijing Computational Science Research Center, Beijing 100193, China
[4]School of Mechanical, Electrical & Information Engineering, Shandong University (Weihai), Weihai 264209, China
[5]Laboratory for Microstructures, Institute of Materials, Shanghai University, Shanghai 200444, China
[6]Erich Schmid Institute of Materials Science, Austrian Academy of Sciences, Jahnstraße 12, A-8700 Leoben, Austria
[7]Department Materials Physics, Montanuniversität Leoben, Jahnstraße 12, A-8700 Leoben, Austria

E-mail: J.G. W. (email: wangjg@seu.edu.cn); Y. P. (email: panye@seu.edu.cn).

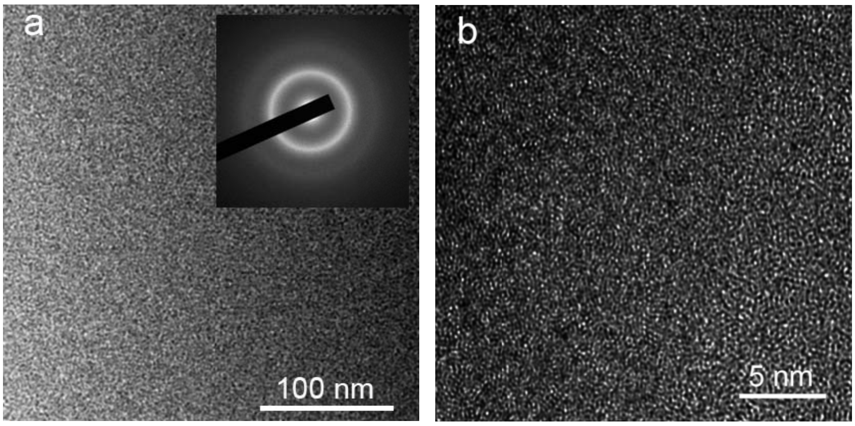

Fig. S1. The investigation on the microstructure in the $Zr_{65}Cu_{15}Ni_{10}Al_{10}$ alloy. (a) The TEM image with SAED pattern (inset) and (b) the HRTEM image. Obviously, the $Zr_{65}Cu_{15}Ni_{10}Al_{10}$ is fully glassy. Extremely similar features are also found for Vit 1 alloy, which is however not shown here.

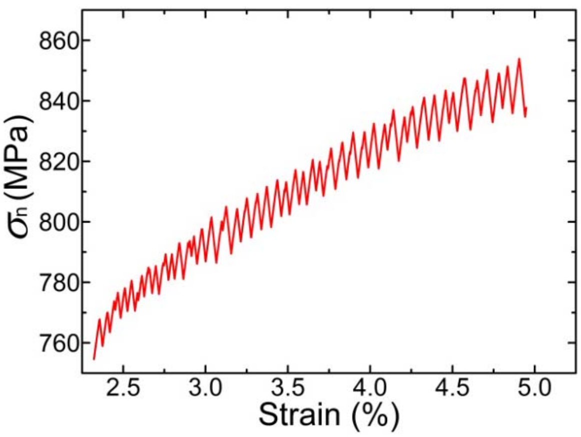

Fig. S2. The normal stress σ_n on the shear plane in Fig.3. One can easily find that σ_n increases with the increasing plastic strain, which will make the effect of hydrostatic pressure greater and greater during the plastic deformation.

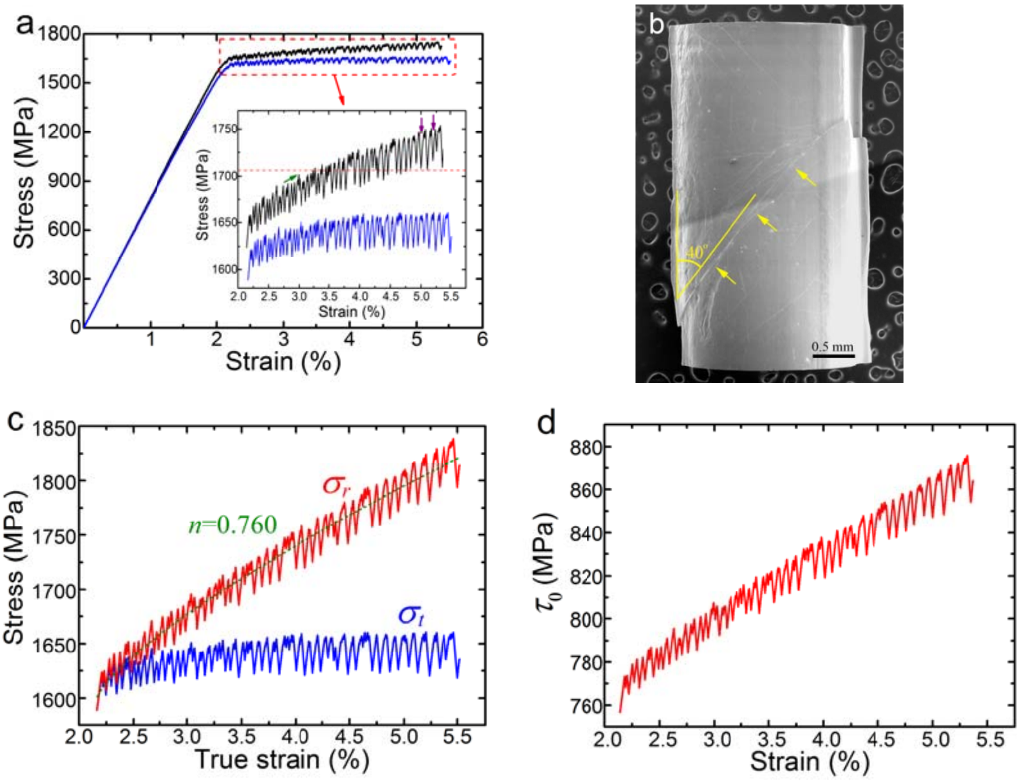

Fig. S3. The investigation on the deformation behavior of $Zr_{65}Cu_{15}Ni_{10}Al_{10}$ MG under compression. (a) Engineering (black) and true (blue) stress-strain curves and the inset magnifying the plastic regime. (b) The SEM image of the deformed sample in which the primary shear band is marked by arrows. (c) The real stress (red) calculated using the area A in Eq.(2) shows the hardenability in $Zr_{65}Cu_{15}Ni_{10}Al_{10}$ MG with a strain-hardening coefficient n=0.760. (d) The intrinsic shear strength calculated by Eq. (4) also shows a hardening behavior.

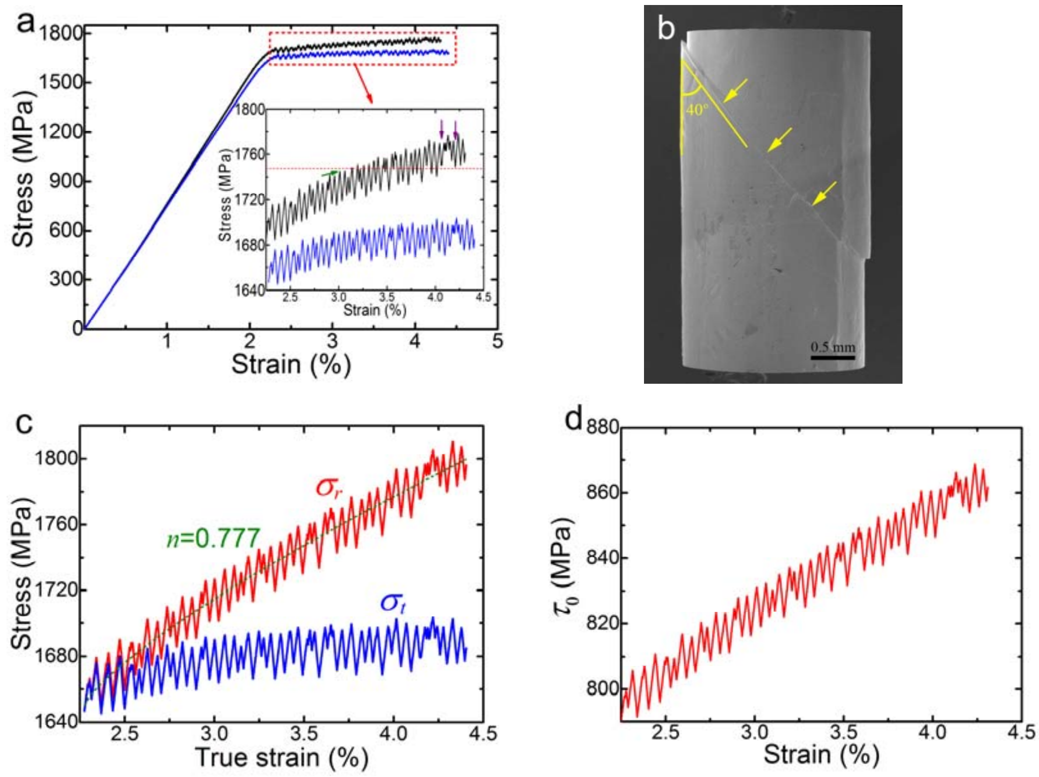

Fig. S4. The investigation on the deformation behavior in Vit 1 MG under compression. (a) The engineering (black) and true (blue) stress-strain curves and the inset magnifying the plastic regime. (b) The SEM image of the deformed sample in which the primary shear band is marked by the arrows. (c) The real stress (red) calculated using the area A in Eq.(2) shows the hardenability in Vit 1 MG with a strain-hardening coefficient n=0.777. (d) The intrinsic shear strength calculated by Eq.(4) also shows a hardening behavior.

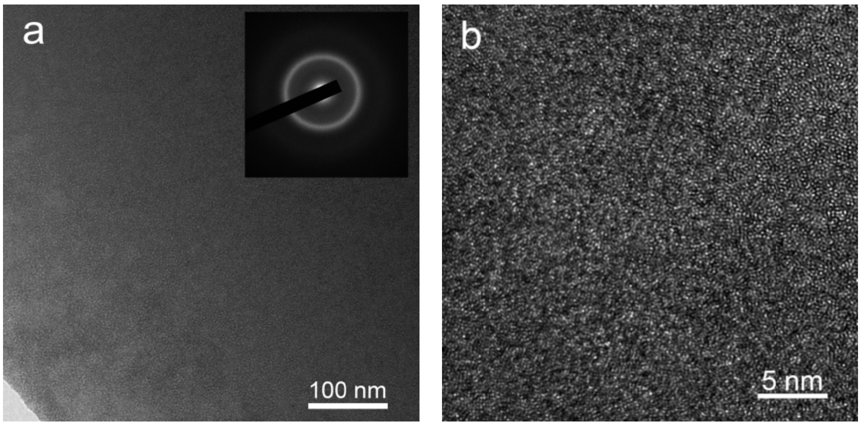

Fig. S5. Microstructure of the deformed $Zr_{50}Cu_{44}Al_6$ sample shown in Fig.3b and Fig.4. (a) The TEM image with SAED pattern (inset) and (b) HRTEM image. Clearly, the microstructure still has a typical feature of amorphous materials.

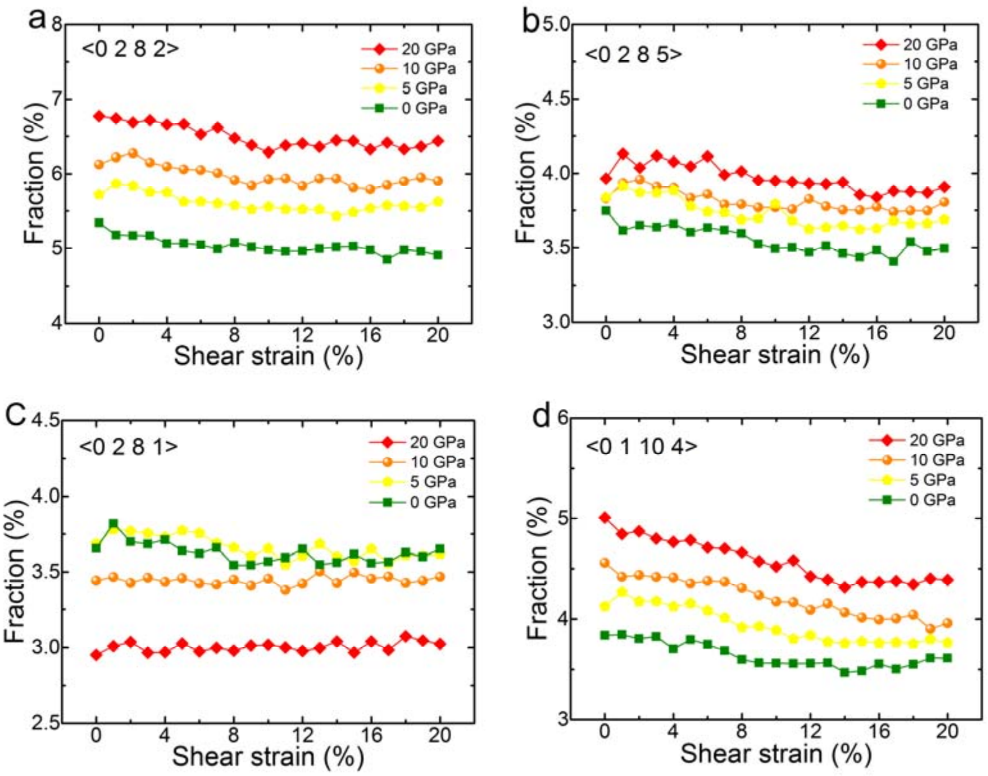

Fig. S6. The fraction of Voronoi polyhedra indexed (a) <0 2 8 2>, (b) <0 2 8 5>, (c) <0 2 8 1> and (d) <0 1 10 4> during the shear deformation of $Zr_{50}Cu_{44}Al_6$ MG samples prepared under different hydrostatic pressures in MD simulations. One can easily find that all the four kinds of polyhedra are affected insignificantly by the shear.
